# Exploratory Integrated Clinical Score and ICF-Defined Functioning in Non-Small Cell Lung Cancer: A Cross-Sectional Study

**DOI:** 10.3390/jcm15218211

**Published:** 2026-10-22

**Authors:** Tania-Ioana Goncea (Pencea), Mara-Amalia Bălteanu, Simona Maria Roșu, Daniela Matei, Magdalena Rodica Trăistaru

**Affiliations:** 1 Doctoral School, University of Medicine and Pharmacy of Craiova, 200349 Craiova, Romaniasimona.rosu@umfcv.ro (S.M.R.); rodica.traistaru@umfcv.ro (M.R.T.); 2 Department of Pneumology, “Marius Nasta” Institute of Pneumophthisiology, 050159 Bucharest, Romania; mara.balteanu@prof.utm.ro; 3 Faculty of Medicine, Titu Maiorescu University, 031593 Bucharest, Romania; 4 Department of Physiotherapy, Faculty of Nursing, University of Medicine and Pharmacy of Craiova, 200349 Craiova, Romania

**Keywords:** non-small cell lung cancer, pulmonary rehabilitation, International Classification of Functioning, Disability and Health, spirometry, systemic inflammation, neutrophil-to-lymphocyte ratio, platelet-to-lymphocyte ratio, functional status

## Abstract

**Background/Objectives**: Spirometry is essential for respiratory assessment but does not fully characterize disability or everyday functioning. This study examined whether an exploratory Integrated Clinical Score (ICS), combining spirometric impairment and systemic inflammation, was associated with selected International Classification of Functioning, Disability and Health (ICF) categories in patients with non-small cell lung cancer (NSCLC) entering pulmonary rehabilitation. **Methods**: This cross-sectional baseline analysis included 62 consecutively recruited adults with histologically confirmed NSCLC. The ICS combined standardized FEV_1_, FVC, FEV_1_/FVC, and natural-log-transformed neutrophil-to-lymphocyte and platelet-to-lymphocyte ratios; higher values indicated greater disease burden. Eleven prespecified ICF categories were rated; eight categories formed the primary analysis set, while three Environmental Factors categories were analysed separately as exploratory. Associations were examined using Spearman correlations with Benjamini–Hochberg correction and proportional-odds models adjusted for age, sex, body mass index, and smoking status. **Results**: Higher ICS values were associated with greater difficulty in energy and drive functions, exercise tolerance, walking, daily routine, housework, and recreation and leisure (ρ = 0.34–0.39; all adjusted *p* < 0.05). Emotional functions were not associated with the ICS; pain reached significance only in the adjusted model (OR 1.61, 95% CI 1.02–2.55). Separate component analyses indicated that these associations were predominantly driven by the lung-function subscore, whereas the inflammation subscore was not significantly associated with any primary ICF category after correction for multiple testing. A 1-SD increase in the ICS was associated with a 0.37-point increase in the summary functional-difficulty index (95% CI 0.13–0.61; *p* = 0.003). **Conclusions**: The exploratory ICS was associated with functioning across several ICF domains, but these associations were predominantly driven by its lung-function component, and incremental value from adding inflammatory markers was not demonstrated. The ICS should therefore be regarded as a hypothesis-generating research construct rather than a validated clinical instrument; further studies should assess its reproducibility and incremental value beyond lung function alone.

## 1. Introduction

Lung cancer develops as a result of uncontrolled growth of abnormal cells in the lungs. It is broadly classified into two major histological types: non-small cell lung cancer (NSCLC), representing nearly 85% of all diagnosed cases, and small cell lung cancer (SCLC), which is less common but characterized by a more aggressive clinical course [1]. Patients with lung cancer commonly experience dyspnea, fatigue, cough, pain and psychological distress, symptoms that often limit exercise tolerance, daily functioning and health-related quality of life [2,3].

The coexistence of lung cancer and COPD is associated with worse clinical outcomes and poses additional challenges for patient management. COPD is one of the most common respiratory comorbidities in patients with lung cancer, with up to 70% of affected individuals presenting both conditions [4]. Therefore, respiratory function should be interpreted within the broader context of chronic respiratory disease rather than as a consequence of the malignancy alone.

Spirometry remains fundamental for identifying and quantifying ventilatory impairment. Its findings should always be interpreted alongside the patient’s clinical condition and the probability of underlying respiratory disease [5,6]. Nevertheless, spirometric variables provide only one perspective on health status and correlate incompletely with dyspnea, exercise limitation, disability, and quality of life. Multidimensional indices such as BODE (this index integrates body mass index, airflow obstruction, dyspnea and exercise capacity), ADO (this index incorporates age, dyspnea and airflow obstruction), and DOSE (this index brings together dyspnea, airflow obstruction, smoking status and exacerbation frequency) were developed to extend COPD assessment beyond airflow obstruction, but their principal purposes are prognostic stratification, disease-severity characterization, or estimation of adverse outcomes rather than a direct description of functioning within a rehabilitation framework [7,8,9].

Systemic inflammation may contribute to reduced physical performance through several mechanisms, including changes in circulating blood cells, cancer-related cachexia, skeletal-muscle catabolism, and reduced muscle strength [10,11]. The neutrophil-to-lymphocyte ratio (NLR) and platelet-to-lymphocyte ratio (PLR) are inexpensive blood-cell-derived markers that have been associated with prognosis in NSCLC [12,13]. In patients with respiratory disease, systemic inflammation may therefore provide information that is complementary to spirometric results, although it does not directly measure functioning.

The American Thoracic Society/European Respiratory Society (ATS/ERS) defines pulmonary rehabilitation as a comprehensive, individualized intervention based on a detailed patient evaluation and targeted therapies designed to improve the physical and psychological status and the health-related quality of life of patients with chronic respiratory diseases [14,15,16]. Because rehabilitation goals extend beyond lung function, assessment should include body functions, activity limitations, participation restrictions, and contextual factors. The World Health Organization International Classification of Functioning, Disability and Health (ICF) provides a standardized biopsychosocial framework for describing these dimensions through an approach that is relevant to rehabilitation [17,18]. The ICF Rehabilitation Set, also known as the ICF Generic-30 Set, contains categories intended to support a broad description of functioning in rehabilitation settings [19,20].

Pulmonary function and systemic inflammatory biomarkers provide information about different aspects of the clinical status of patients with NSCLC. Spirometry remains essential for characterizing ventilatory impairment but, when interpreted in isolation, does not fully reflect disability, quality of life, dyspnea, or overall health status [21]. Conversely, systemic inflammatory markers may reflect biological processes associated with cancer-related and respiratory disease burden that are not directly captured by spirometry. NLR and PLR are readily available and cost-effective blood-based markers of systemic inflammation [22]. These domains may therefore be considered potentially complementary rather than interchangeable. On this basis, the ICS was developed as an exploratory analytical construct to summarize routinely available spirometric and inflammatory information within a single measure and to examine whether their combined profile was associated with rehabilitation-relevant functional difficulty. The purpose of combining these domains was not to assume or establish superiority over conventional spirometry, but to explore whether integrating distinct physiological and inflammatory information could provide a broader representation of clinical burden in relation to ICF-defined functioning. Whether the inflammatory component provides information beyond lung function alone was therefore considered an empirical question rather than an assumption underlying the score. Accordingly, the ICS should not be regarded as a replacement for direct clinical assessment or validated patient-reported and performance-based outcomes. The primary objective of this study was to examine the association between an exploratory Integrated Clinical Score (ICS), combining spirometric impairment (FEV_1_, FVC and the FEV_1_/FVC ratio were selected because they provide complementary information on the severity of airflow limitation, potential ventilatory restriction and the presence of expiratory airflow obstruction [23]) and systemic inflammation (NLR and PLR), and 11 prespecified categories from the ICF Rehabilitation Set in patients with NSCLC entering pulmonary rehabilitation. Eight of these categories, drawn from the ICF Rehabilitation Set (also referred to as the ICF Generic-30 Set) and selected for their clinical relevance to rehabilitation, formed the primary analysis set; the three Environmental Factors categories were examined separately as exploratory analyses. We hypothesized that higher ICS values, indicating poorer lung function and greater systemic inflammation, would be associated with higher ICF qualifier scores. Secondary objectives were to assess the relationship between the lung-function and inflammation subscores, to examine the association of each subscore with the ICF categories separately in order to identify which component accounted for any association of the composite, and to examine whether the associations persisted after adjustment for age, sex, body mass index and smoking status.

## 2. Materials and Methods

### 2.1. Study Design, Setting, and Ethics

This study presents a cross-sectional analysis of baseline data from a prospectively enrolled observational cohort of patients undergoing pulmonary rehabilitation at the “Marius Nasta” Institute of Pneumophthisiology, Bucharest, Romania. Following ethics approval, participants were evaluated in the course of routine rehabilitation care. The present analysis concerns measurements obtained before the rehabilitation program and does not evaluate treatment effectiveness. Participants were recruited consecutively and evaluated during the approved study period, with data collection completed in September 2024. Reporting follows the Strengthening the Reporting of Observational Studies in Epidemiology (STROBE) recommendations for cross-sectional studies, and the completed checklist is provided as Supplementary Material.

The study was conducted in accordance with the Declaration of Helsinki and was approved by the Ethics Committee of the “Marius Nasta” Institute of Pneumophthisiology (approval no. 23361/17 October 2023) and the Ethics Committee of the University of Medicine and Pharmacy of Craiova (approval no. 91/19 February 2024). All participants provided written informed consent before inclusion.

Evaluation using the ICF Rehabilitation Set (also referred to as the ICF Generic-30 Set) was performed at baseline. This non-invasive assessment did not interfere with routine clinical care, preserving the observational nature of the study.

The research team was granted authorized access to the medical records and study-related data required for the analyses.

### 2.2. Participants and Eligibility Criteria

Consecutive patients meeting the eligibility criteria were included throughout the study period. Adults aged ≥18 years were eligible if they were Romanian residents, had histologically confirmed lung adenocarcinoma or squamous cell carcinoma (established by histopathological examination of bronchoscopic biopsy specimens and classified according to the WHO Classification of Thoracic Tumours), had a clinical indication for pulmonary rehabilitation, could understand and follow rehabilitation instructions, and were able to attend the scheduled evaluations by the multidisciplinary team.

Patients were excluded if cognitive impairment prevented informed consent or adherence to instructions; if they had thromboembolic disease, unstable cardiovascular disease, severe orthopedic or post-traumatic conditions, rheumatic disease affecting mobility or upper-limb function, neuromotor disease interfering with locomotion, or another clinically relevant condition considered likely to substantially influence respiratory function, exercise capacity, or interpretation of the study findings.

**Figure 1:**
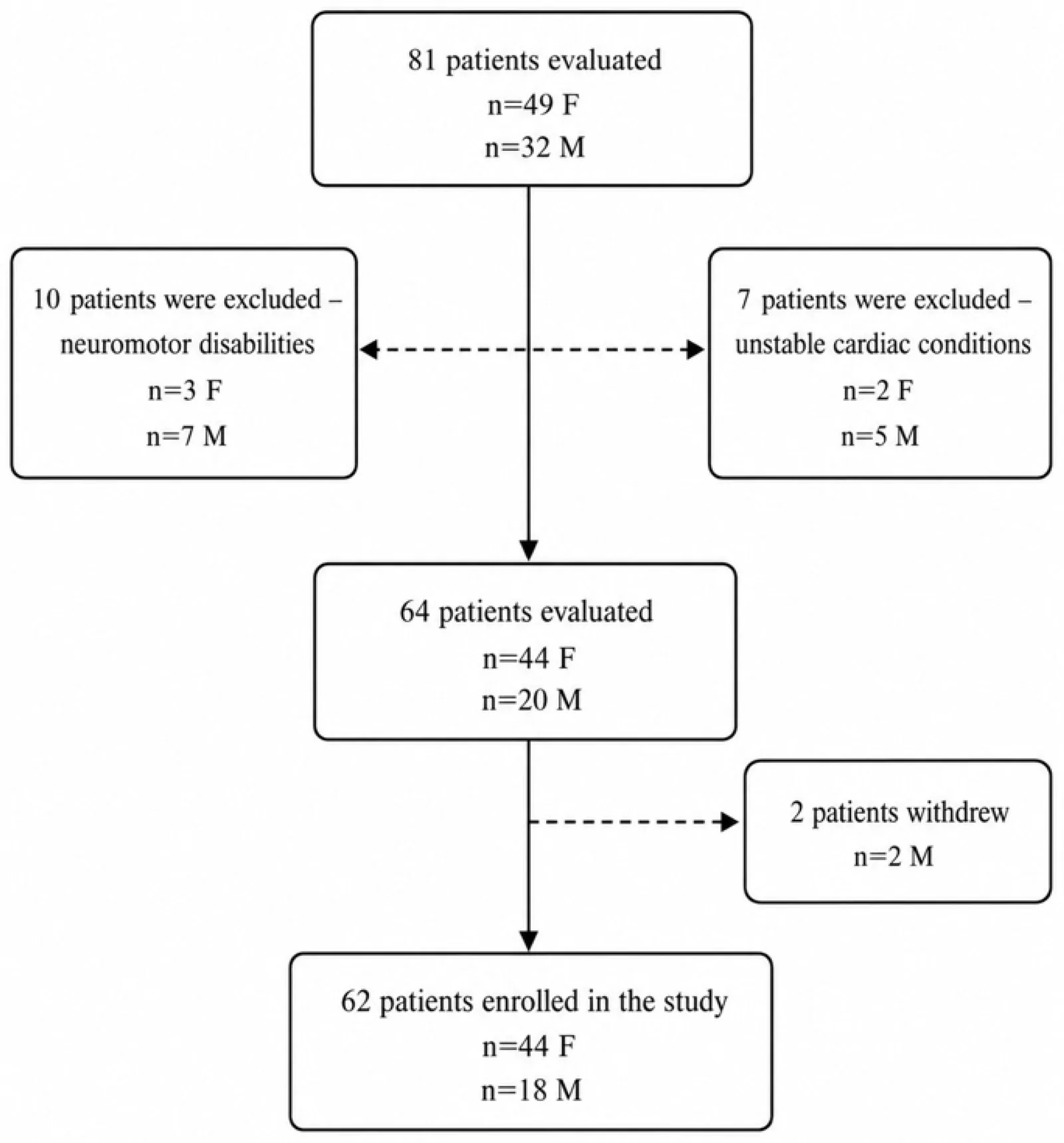
Participant flow diagram. F = female; M = male.

Clinically documented COPD was identified from the medical record. Where available, the diagnosis had been established in routine clinical care in accordance with contemporaneous Global Initiative for Chronic Obstructive Lung Disease (GOLD 2025) recommendations [16], which require persistent post-bronchodilator airflow obstruction (FEV_1_/FVC < 0.70) in the appropriate clinical context. Because post-bronchodilator spirometry was not systematically available in the study dataset, baseline pre-bronchodilator spirometry was not used to establish a COPD diagnosis. Pre-bronchodilator airflow obstruction, defined as a pre-bronchodilator FEV_1_/FVC ratio < 0.70, was present in 41 of the 62 participants (66.1%). Because post-bronchodilator spirometry was not systematically available, this finding was reported separately from clinically documented COPD and was not considered sufficient to establish a COPD diagnosis. The participant selection process is shown in the flowchart (Figure 1). Patients were entering an eight-week individualized pulmonary rehabilitation program delivered within multidisciplinary care. The program included supervised aerobic and respiratory training, supported by home exercises and, when clinically indicated, psychological and nutritional counselling. The present cross-sectional analysis used only baseline data collected before pulmonary rehabilitation and did not evaluate treatment effectiveness.

### 2.3. Baseline Clinical and Laboratory Assessment

Baseline data were collected before the start of pulmonary rehabilitation. Demographic and clinical variables comprising age, sex, body mass index (BMI), smoking status and peripheral oxygen saturation (SpO_2_) while breathing room air were extracted from the observation sheets completed during the initial evaluation (Table 1). Cancer stage and current or recent oncologic treatment status were not documented with sufficient consistency across medical records to support reliable tabulation or covariate analysis and were therefore not included in the analytical dataset.

Age, sex, height, and ethnic group were entered into the spirometry software for calculation of FEV_1_ and FVC percent-predicted values according to the GLI-2012 reference equations. BMI and smoking status were recorded as clinical characteristics but were not inputs to the GLI-2012 prediction equations; they were used as covariates in the adjusted statistical models. We considered age, sex, BMI, and smoking status clinically relevant because these routinely available characteristics may contribute to between-patient differences in functional status. Current smoking and female sex have been associated with poorer physical performance and less favorable functional outcomes at baseline in patients undergoing pulmonary and cardiac rehabilitation [24]. Advancing age is associated with a decline in the physical domain of health-related quality of life, with comorbidities, physical activity and lifestyle [25]. Systemic inflammatory status was evaluated using the neutrophil-to-lymphocyte ratio (NLR) and platelet-to-lymphocyte ratio (PLR). Both ratios were calculated from the complete blood count available at baseline (T0). NLR was calculated by dividing the absolute neutrophil count by the absolute lymphocyte count, and PLR by dividing the platelet count by the absolute lymphocyte count.

Histopathological confirmation in all participants was based on tissue samples collected during flexible bronchoscopy. Depending on the lesion’s location and bronchoscopic accessibility, samples were collected through endobronchial or transbronchial biopsy. The tissue was fixed in 10% neutral buffered formalin, processed into paraffin blocks, and sectioned at a thickness of 3–5 μm for microscopic examination.

### 2.4. Spirometry

Spirometry was performed using a Vyntus SPIRO system (Jaeger Medical GmbH, Höchberg, Germany) with SentrySuite software version 2.21, in a seated position with a nose clip, in accordance with the 2019 ATS/ERS technical standard [26]. Forced expiratory volume in 1 s (FEV_1_), forced vital capacity (FVC), and the FEV_1_/FVC ratio were recorded at baseline, before pulmonary rehabilitation, and used to characterize pulmonary function. FEV_1_ and FVC percent-predicted values were calculated by the spirometry software using the Global Lung Function Initiative 2012 (GLI-2012) reference equations, which account for age, sex, height, and ethnic group and are applicable across the 3–95-year age range; all study participants were within this range. At least three acceptable forced expiratory maneuvers were obtained, and measurements were considered repeatable when the difference between the two largest FEV_1_ values and between the two largest FVC values did not exceed 150 mL.

Coexisting COPD was identified from the medical record when available. Baseline spirometry was used to describe ventilatory patterns, including pre-bronchodilator airflow obstruction, but post-bronchodilator confirmation was not available systematically. A reduced FVC with a preserved FEV_1_/FVC ratio was interpreted as a spirometric pattern suggestive of restriction rather than as confirmed restrictive ventilatory impairment, whereas a reduced FVC combined with an FEV_1_/FVC ratio < 0.70 was described as a mixed spirometric pattern for descriptive purposes only and was not interpreted as confirmation of a mixed obstructive–restrictive ventilatory defect.

### 2.5. ICF-Based Functional Assessment

To outline the rehabilitation-related profile of the study group, eleven categories were selected and scored—eight from the ICF Rehabilitation Set (also referred to as the ICF Generic-30 Set), comprising four Body Functions and four Activities and Participation categories, together with three categories from the companion minimal set of Environmental Factors (Table 2). The eight categories included in the primary analysis were selected before examination of their associations with the ICS, as part of the baseline functional assessment of patients entering pulmonary rehabilitation. Selection was based on their clinical relevance to rehabilitation and was intended to represent complementary aspects of Body Functions and Activities and Participation, including energy and drive, emotional functions, pain, exercise tolerance, daily routine, walking, housework, and recreation and leisure. The selection was not based on the magnitude or statistical significance of their subsequent associations with the ICS. The three Environmental Factors categories were analysed separately as exploratory outcomes. The assessment was completed at baseline by a trained physical therapist who was part of the multidisciplinary rehabilitation team.

For the Body Functions and Activities and Participation categories, the standard ICF 0–4 problem qualifier was used, where 0 indicates no problem and 4 indicates a complete problem; codes 8 (“not specified”) and 9 (“not applicable”) were treated as missing. For the three Environmental Factors categories, an analogous study-defined 0–4 problem-severity rating was applied for analytical consistency rather than the standard ICF barrier/facilitator qualifier. This study-defined rating captured the severity of the problem experienced and preserved a common 0–4 outcome metric across the analysed categories; however, it should not be interpreted as equivalent to standard ICF qualification of Environmental Factors as barriers or facilitators. Missingness affected d920 (*n* = 1), e110 (*n* = 2), e115 (*n* = 31), and e120 (*n* = 43), while the remaining categories were complete. Table 3 shows the rating levels and their interpretation.

### 2.6. Construction of the Integrated Clinical Score

The ICS was constructed from two prespecified domains: lung function and systemic inflammation.

The two inflammatory ratios, NLR and PLR, were natural-log-transformed before standardization to correct their pronounced right-skew (skewness fell from 1.45 to 0.13 for NLR and from 1.59 to 0.32 for PLR, approximating normality) and then standardized as z-scores. The inflammation subscore was calculated as the mean of the z-scores of ln (NLR) and ln (PLR), with higher values denoting greater systemic inflammation.

A lung-function subscore was computed as the arithmetic mean of the z-scores of FEV_1_ (% predicted), FVC (% predicted), and FEV_1_/FVC, with higher values denoting better lung function. These variables were retained because they describe complementary aspects of ventilatory impairment; however, they are physiologically and mathematically related, and averaging standardized variables does not eliminate this dependence. Their mutual correlations were therefore examined explicitly, and sensitivity analyses were undertaken to assess whether the resulting composite was disproportionately influenced by any single component. To give the two domains equal weight, each subscore was re-standardized; the sign of the lung-function subscore was reversed, and the two standardized domains were averaged. Thus, higher ICS values indicated poorer lung function combined with greater systemic inflammation. Equal weighting was selected a priori as a simple and transparent exploratory analytical approach because no established empirical or clinically validated basis was available for assigning greater weight to either domain. This weighting scheme should therefore not be interpreted as empirically derived or validated, and its robustness was examined in sensitivity analyses using alternative domain weights.

ICS = {−z(Lung-function subscore) + z(Inflammation subscore)}/2.

**Figure 2:**
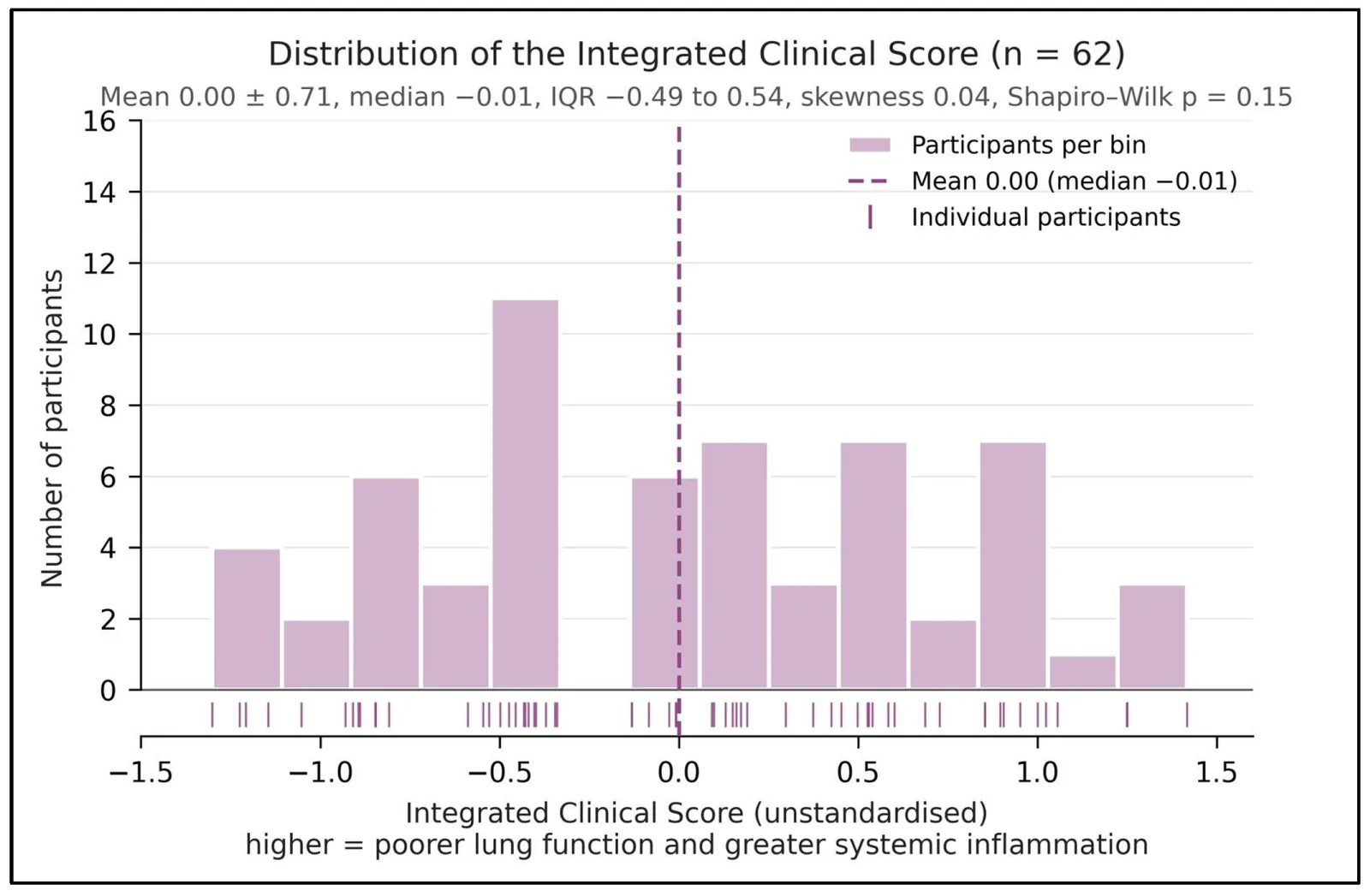
Distribution of the Integrated Clinical Score across the 62 participants.

For all regression models the averaged ICS was itself re-standardised to mean 0 and standard deviation 1, so that regression coefficients and odds ratios are expressed per 1-SD increase; the descriptive statistics in 3.2 and Figure 2 report the unstandardised composite, whose standard deviation is 0.71. Accordingly, a 1-SD increase in the standardized ICS represents an increase of one standard deviation relative to the score distribution in the study cohort, rather than a one-unit increase in the original composite score. Regression estimates should therefore be interpreted as the change in the outcome, or in the odds of greater ICF-defined difficulty, associated with this relative increase in overall ICS burden. As the ICS is an exploratory and non-validated construct, no clinically meaningful threshold or cut-off value has yet been established, and the score should currently be interpreted as a continuous research measure rather than as a clinical classification tool.

### 2.7. Statistical Analysis

Statistical analyses were performed using Python 3.13.5 (Python Software Foundation, Wilmington, DE, USA), together with the SciPy 1.17.0 and statsmodels 0.14.6 libraries. No formal a priori sample-size calculation was performed. The sample comprised all eligible patients recruited consecutively during the study period. Given the exploratory nature of the study and the fixed sample size, statistical interpretation focused on effect estimates and their 95% confidence intervals rather than on retrospective power calculations.

Continuous variables were summarized using mean and standard deviation or median and interquartile range, according to distribution; categorical variables were summarized using counts and percentages. The distribution of the ICS was evaluated graphically and using an appropriate normality assessment.

The primary association between the ICS and each ICF category was estimated using Spearman’s rank correlation coefficient because the ICF qualifiers are ordinal. The eight Body Functions and Activities and Participation categories constituted the primary analysis set, and *p* values from these eight tests were adjusted using the Benjamini–Hochberg procedure, with a two-sided adjusted *p* < 0.05 considered statistically significant; the three Environmental Factors categories were analysed separately as exploratory and corrected within that set (Table S4). Pearson coefficients were computed in parallel for comparison. Confidence intervals for Spearman coefficients were obtained by percentile bootstrap with 5000 resamples.

Each ICF category was additionally analyzed using proportional-odds ordinal logistic regression. Adjusted models included age, sex, BMI, and smoking status; smoking status was entered as a three-level categorical variable (current smoker, former smoker, and never smoker), with never smoker used as the reference category. The proportional-odds assumption and the distribution of observations across qualifier levels were examined for each ordinal outcome. Because the adjusted models were fitted in a modest cohort and some qualifier levels were sparsely populated, model stability and precision were considered when interpreting the estimates; the adjusted ordinal analyses were therefore treated as exploratory and supportive rather than definitive. A summary functional-difficulty index was calculated as the mean of the eight Body Functions and Activities and Participation qualifiers; environmental-factor categories were excluded from this index. Missing values were handled by pairwise deletion, so each estimate was computed on all patients with a valid value for that category. Peripheral oxygen saturation was examined in a pre-specified sensitivity model added to the fully adjusted linear model. Because all eligible patients presenting during the recruitment period were enrolled consecutively at a single centre, no stratification, clustering or sampling weights applied, and no design-based adjustment was required. Additional sensitivity analyses examined the robustness of the ICS construction. First, Spearman and Pearson correlations among FEV_1_% predicted, FVC % predicted, FEV_1_/FVC, ln (NLR), and ln (PLR), as well as among the blood-cell counts underlying NLR and PLR, were calculated to characterize dependence among score components. Second, each of the five ICS components was omitted in turn, and the remaining components within that domain were averaged and re-standardized using the same procedure as for the primary score. Third, to address the shared lymphocyte denominator of NLR and PLR, the inflammation subscore was alternatively reconstructed from the z-scores of ln (neutrophil count), ln (platelet count), and sign-reversed ln (lymphocyte count), so that each blood-cell count entered only once. Fourth, the relative weighting of the lung-function and inflammatory domains was varied from 100/0 to 0/100 (100/0, 75/25, 67/33, 50/50, 33/67, 25/75, and 0/100). Each alternative score was re-standardized and analyzed using the same procedures as the primary ICS, including Spearman correlations with the eight primary ICF categories, Benjamini–Hochberg correction within each score specification, and the adjusted linear model for the summary functional-difficulty index. These sensitivity analyses were exploratory and were not prespecified; all results are reported in Section 3.5 and Table S6.

Analyses were performed in Python 3.13 using SciPy 1.16 for rank correlations and distributional tests, statsmodels 0.15.1 for proportional-odds ordinal regression, linear regression and Benjamini–Hochberg false-discovery-rate control, and pandas 2.2 and NumPy 2.1 for data handling. All statistical tests were two-sided.

## 3. Results

### 3.1. Participant Characteristics

Of 81 patients initially considered for inclusion, 17 were excluded (10—3 F, 7M—were excluded because of neuromotor disorders limiting locomotion, and 7—2 F, 5 M—because of unstable cardiac conditions that made rehabilitation unsafe) and 2 (2 M) withdrew before baseline assessment; 62 participants (44 F, 18 M) completed the baseline evaluation and were included in the analysis (Figure 1) and began the rehabilitation program. The mean age was 69.9 ± 8.87 years, and 44 participants (71.0%) were women. Current smokers accounted for 38.7% of the cohort, former smokers for 41.9%, and never smokers for 19.4%. Lung adenocarcinoma was present in 33 participants (53.2%) and squamous cell carcinoma in 29 (46.8%). The most frequent spirometric pattern was mixed (21 patients, 33.9%), followed by obstructive (20, 32.3%), a spirometric pattern suggestive of restriction (12, 19.4%), and normal (9, 14.5%). Pre-bronchodilator airflow obstruction (FEV_1_/FVC <0.70) was present in 41 patients (66.1%) and was reported separately from clinically documented COPD. Baseline characteristics are summarized in Table 1.

### 3.2. Distribution of the ICS and Relationship Between Subscores

The Integrated Clinical Score was approximately normally distributed (mean 0.00 ± 0.71, median −0.01, IQR −0.49 to 0.54, range −1.30 to 1.42, skewness 0.04; Shapiro–Wilk *p* = 0.15; Figure 2). Its components were also centered and symmetric: the lung-function subscore had a mean of 0.00 ± 0.87, and the inflammation subscore had a mean of 0.00 ± 0.92.

The lung-function and inflammation subscores were not significantly correlated (Spearman *ρ* = −0.05; *p* = 0.715; 95% bootstrap confidence interval −0.28 to 0.20). This absence of correlation suggests that the two domains captured different aspects of the patients’ clinical profile; however, it does not establish statistical independence or demonstrate incremental predictive value of the combined score. Examined separately, the lung-function subscore was associated with greater difficulty in the same categories (*ρ* = −0.29 to −0.43 among those reaching significance), whereas the inflammation subscore was not significantly associated with any ICF category after correction for multiple testing (*ρ* = −0.10 to 0.18 across the eight primary categories; smallest Benjamini–Hochberg-adjusted *p* = 0.47). These findings do not demonstrate added value from combining the inflammatory and lung-function domains (Table S1).

### 3.3. Unadjusted Associations Between the ICS and ICF Categories

**Figure 3:**
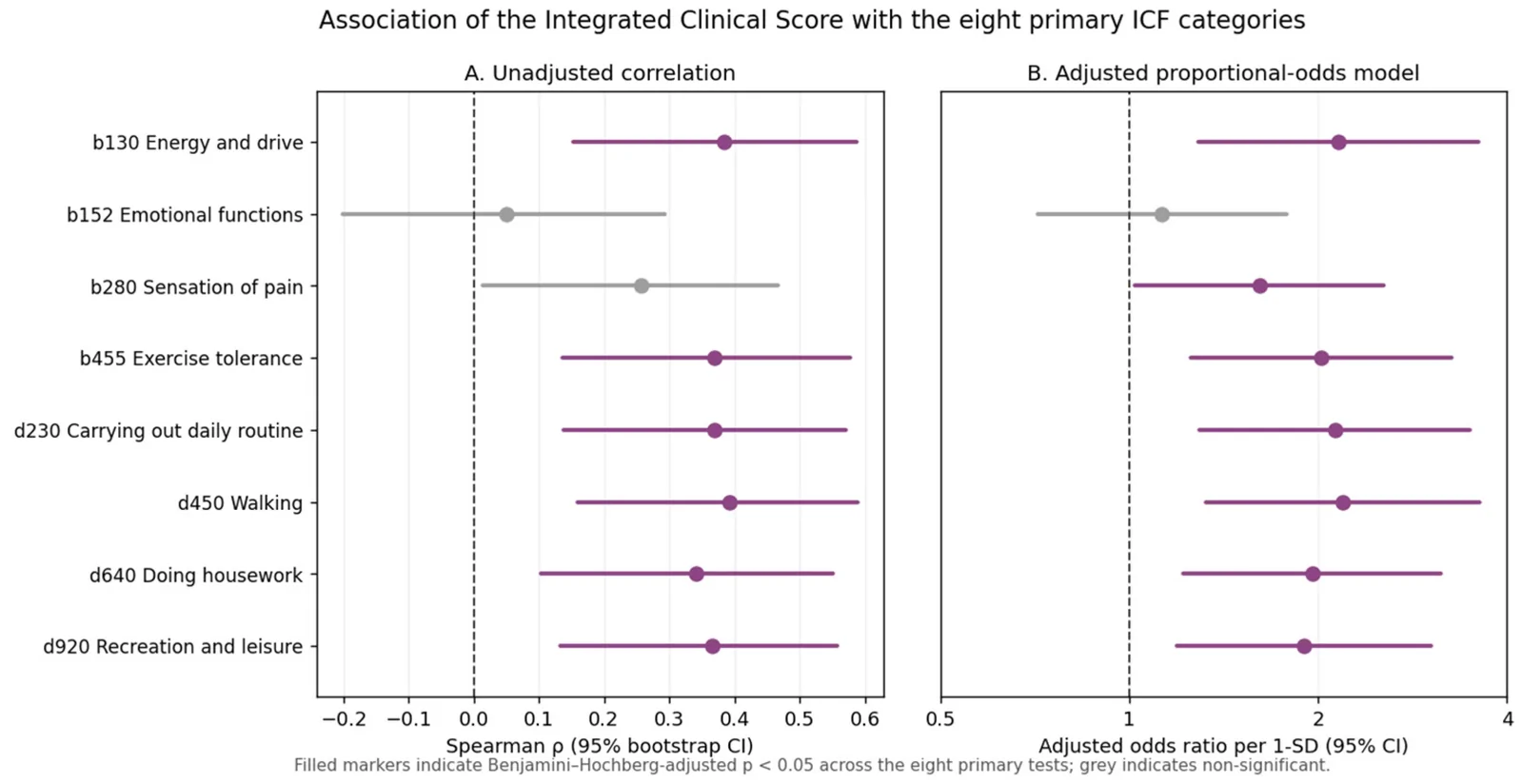
Association of the Integrated Clinical Score with the eight primary ICF categories. (**A**) Spearman correlation coefficients with 95% bootstrap confidence intervals (5000 resamples). (**B**) Odds ratios per 1-SD increase in the ICS from proportional-odds models adjusted for age, sex, BMI, and smoking status, with 95% confidence intervals. Coloured markers indicate Benjamini–Hochberg-adjusted *p* < 0.05 across the eight primary tests; grey markers indicate non-significant associations. Sensation of pain (b280) reached statistical significance in the adjusted model only. For d640 and d920, the proportional-odds assumption was not satisfied; therefore, their common odds ratios are presented for descriptive purposes only and should not be interpreted as constant effects across qualifier thresholds. Threshold-specific estimates are provided in Table S5.

The eight Body Functions and Activities and Participation categories formed the primary analysis set, and Benjamini–Hochberg correction was applied across these eight tests; the three Environmental Factors categories are reported separately as exploratory in Supplementary Materials. Higher ICS values were significantly associated with greater difficulty in six of the eight primary categories (Table 4, Figure 3). Within Body Functions, significant associations were observed for energy and drive functions (b130; *ρ* = 0.38; adjusted *p* = 0.006) and exercise tolerance functions (b455; *ρ* = 0.37; adjusted *p* = 0.006). Emotional functions were unrelated to the ICS (b152; ρ = 0.05; adjusted *p* = 0.701), and sensation of pain fell just short of significance (b280; *ρ* = 0.26; adjusted *p* = 0.050).

Within Activities and Participation, the ICS was associated with walking (d450; *ρ* = 0.39; adjusted *p* = 0.006), carrying out the daily routine (d230; ρ = 0.37; adjusted *p* = 0.006), doing housework (d640; ρ = 0.34; adjusted *p* = 0.009), and recreation and leisure (d920; *ρ* = 0.37; adjusted *p* = 0.006; *n* = 61).

Results for the three Environmental Factors categories are presented as exploratory in Table S4 rather than in the primary analysis, because the high frequency of “not applicable” responses resulted in substantially smaller and selected analytic samples, particularly for e115 (*n* = 31) and e120 (*n* = 19). Consequently, these estimates should not be interpreted as directly comparable with those obtained for the primary ICF categories; the implications of this missingness are discussed in Section 4.4.

### 3.4. Adjusted Associations

In the multivariable linear model, a 1-SD increase in the ICS was associated with a 0.37-point higher summary functional-difficulty index after adjustment for age, sex, BMI, and smoking status (95% CI 0.13–0.61; *p* = 0.003; *R*^2^ = 0.19). Adjusted ordinal regression results for the individual ICF categories are presented in Table 5.

For outcomes for which the proportional-odds assumption was adequately supported, each 1-SD increase in the ICS was associated with higher odds of being in a worse qualifier category for energy and drive functions (adjusted odds ratio [OR], 2.16; 95% CI 1.29–3.61), exercise tolerance (OR, 2.02; 1.25–3.26), walking (OR, 2.19; 1.33–3.62), carrying out the daily routine (OR, 2.13; 1.29–3.49), and sensation of pain (OR, 1.61; 1.02–2.55). The common ORs for doing housework (d640; OR, 1.96; 1.22–3.14) and recreation and leisure (d920; OR, 1.90; 1.19–3.03) are reported descriptively only because the proportional-odds assumption was not satisfied. Emotional functions remained unassociated with the ICS (OR, 1.13; 0.71–1.78; adjusted *p* = 0.606). The association with pain reached statistical significance only in the adjusted ordinal model, whereas the Benjamini–Hochberg-adjusted *p* value in the Spearman correlation analysis was 0.0502. Given this borderline finding, the association with pain should be interpreted cautiously. The proportional-odds assumption was examined for each ordinal outcome. Formal testing was limited by sparse qualifier levels; among the primary categories for which the assumption could be formally assessed, evidence of departure from proportional odds was observed for d640 (doing housework; *p* = 0.006) and d920 (recreation and leisure; *p* = 0.046). Accordingly, no inferential interpretation is assigned to the common odds ratios for d640 and d920; threshold-specific estimates are provided in Table S5 to show how the association varied across qualifier cut-points. Given the modest sample size and sparse qualifier levels, a more complex partial proportional-odds or multinomial model was not fitted because such models would be at substantial risk of instability.

In exploratory post hoc interaction analyses, no statistically significant interaction was detected for sex (*p* = 0.573), smoking status (*p* = 0.543), histological subtype (*p* = 0.224), or airflow obstruction (*p* = 0.727); stratified estimates were directionally consistent across subgroups, and these analyses should be interpreted descriptively (Table S3). In the pre-specified sensitivity model, adding peripheral oxygen saturation attenuated the ICS coefficient to 0.15 (95% confidence interval, −0.08 to 0.38; *p* = 0.196). This attenuation may reflect over-adjustment, because SpO_2_ is itself a marker of pulmonary impairment and is correlated with the lung-function subscore (*r* = 0.47, both oriented so that higher values indicate better pulmonary status); however, the present cross-sectional analysis cannot distinguish over-adjustment from residual confounding.

### 3.5. Sensitivity Analyses of the ICS Construction

Correlations among the ICS components were strong within each domain and negligible between domains. Among the spirometric variables, FEV_1_ correlated with FVC (Spearman ρ = 0.84) and with FEV_1_/FVC (ρ = 0.67), and FVC correlated with FEV_1_/FVC (ρ = 0.38). ln(NLR) and ln(PLR) correlated at ρ = 0.71, whereas no spirometric component correlated with an inflammatory component beyond |ρ| = 0.12. The correlation between NLR and PLR was approximately twice that between their numerators (neutrophil and platelet counts, ρ = 0.34), consistent with a substantial contribution of the shared lymphocyte denominator; in standardized terms, lymphocyte count accounted for 53% of the weight of the inflammation subscore, compared with 25% for neutrophils and 22% for platelets. Omitting any single component left the associations essentially unchanged: the five leave-one-out scores correlated with the primary ICS at *r* = 0.96–0.99, the same six primary ICF categories remained significant in each version, and the adjusted coefficient for the summary functional-difficulty index ranged from 0.35 to 0.39 per 1-SD increase (all *p* ≤ 0.007). For the spirometric components, this stability partly reflects their mutual dependence, because much of the information in an omitted variable is retained by the other two. Reconstructing the inflammation subscore so that each blood count was entered once also left the results essentially unchanged: the alternative score correlated with the primary ICS at *r* = 0.95, the same six primary categories remained significant, and the adjusted coefficient for the summary functional-difficulty index was 0.38 (95% CI 0.14–0.62; *p* = 0.003), compared with 0.37 (95% CI 0.13–0.61; *p* = 0.003) for the primary ICS. Across the weighting grid, associations were stable from 100/0 to 33/67 (lung function/inflammation; adjusted *β* = 0.31–0.41 per 1-SD, all *p* ≤ 0.015; six or seven of the eight primary categories significant) and weakened as more weight was assigned to inflammation. At 25/75, the adjusted coefficient was 0.28 (95% CI 0.02–0.53; *p* = 0.032) and no category remained significant after correction; the inflammation subscore alone was not associated with the summary index (*β* = 0.18; 95% CI −0.09 to 0.44; *p* = 0.186). The equal-weight ICS therefore lies within the range of specifications over which the observed associations were stable, while the overall pattern indicates that these associations were driven predominantly by the lung-function component (Table S6).

## 4. Discussion

### 4.1. Principal Findings

In this cross-sectional baseline analysis of patients with histologically confirmed NSCLC entering pulmonary rehabilitation, an exploratory Integrated Clinical Score (ICS), combining altered spirometric results and systemic inflammation, was associated with functional difficulty across several ICF domains. Higher ICS values were related to greater difficulty in energy and drive functions, exercise tolerance, walking, carrying out the daily routine, doing housework, and recreation and leisure. The unadjusted Spearman analyses showed a consistent direction of association across these domains. Adjusted proportional-odds estimates provided supportive evidence for outcomes in which the model assumption was adequately supported; for d640 and d920, the adjusted ordinal estimates were retained for descriptive purposes only because the proportional-odds assumption was violated. The summary functional-difficulty index also was higher in patients with higher ICS values, supporting a consistent link between the pulmonary–inflammatory profile as summarised by the ICS and limitations across body functions, activities and participation. Although separate analyses of the lung-function and inflammation subscores were performed, no formal assessment of the incremental value of the complete ICS over its individual components was undertaken. Therefore, these findings should not be interpreted as demonstrating an advantage of combining the two domains over lung function alone.

The observed correlations were modest to moderate (ρ = 0.34–0.39), indicating that the ICS accounted for only part of the variability in ICF-defined functioning, which is inherently multifactorial. Because clinically meaningful thresholds for the ICS have not been established and no prospective outcome was evaluated, the clinical significance of these associations cannot yet be determined. No significant association was found for emotional functions, either before or after adjustment, indicating that the ICS was not associated with all dimensions of functioning. Pain was associated with the ICS only in the adjusted model and should therefore be interpreted cautiously given its multidimensional nature [27].

The lung-function and inflammation subscores were not significantly correlated; however, this does not establish statistical independence or demonstrate incremental value from combining the two domains.

### 4.2. Interpretation in Relation to Previous Research

The present findings are consistent with the multidimensional approach to pulmonary rehabilitation, in which spirometry is interpreted alongside symptoms, exercise capacity, health-related quality of life, and participation [15]. This is particularly relevant in NSCLC, where respiratory impairment may coexist with cancer-related fatigue, treatment effects, nutritional decline, and reduced physical activity. Unlike established multidimensional indices such as BODE, ADO, and DOSE, which were developed primarily for prognostic or disease-severity assessment in COPD [7,8,9], the ICS was constructed as an exploratory measure to examine the relationship between routinely available spirometric and inflammatory information and ICF-defined functioning. The present findings do not establish that the ICS is superior to these indices, individual spirometric measures, or direct functional assessment. Previous studies have associated NLR and PLR with lung function, exercise capacity, and disease-related quality of life in COPD, while systemic inflammation in NSCLC has been linked to processes including muscle catabolism and fatigue [11,28]. In the present study, however, the inflammation subscore was not significantly associated with any primary ICF category after correction for multiple testing, and the associations of the ICS were predominantly attributable to the lung-function component. Thus, these findings do not demonstrate an independent contribution of the inflammatory component to ICF-defined functioning. Previous ICF-based investigations also support assessing functioning separately from lung function alone. Li et al. reported limited associations of FEV_1_ and FVC with quality of life after lung surgery, whereas exercise-related and personal factors contributed more substantially [29]. Zawadzka-Fabijan et al. used an ICF framework to characterize postoperative functioning in patients with early-stage NSCLC undergoing pulmonary rehabilitation [30], while Wang et al. identified distinct functional profiles among patients with COPD referred for rehabilitation [31]. The present study extends this perspective by examining associations between routinely available physiological and inflammatory measures and selected ICF categories at rehabilitation entry.

### 4.3. Biological Context for the Observed Associations

The association of the ICS with energy and drive, exercise tolerance, and walking is biologically plausible. Reduced FEV1, FVC, and FEV1/FVC may reflect airflow obstruction, reduced ventilatory reserve, or a spirometric pattern suggestive of restriction; however, restrictive ventilatory impairment cannot be confirmed without lung-volume measurements. These abnormalities may increase ventilatory demand and symptom burden associated with activity. When respiratory limitation is combined with systemic inflammation, patients may experience greater fatigue and reduced capacity to sustain physical effort. This may be especially relevant in the present cohort because pre-bronchodilator airflow obstruction was present in approximately two thirds of patients; this finding should not be interpreted as equivalent to clinically documented COPD.

Systemic inflammation has been linked biologically to skeletal-muscle dysfunction in lung cancer and chronic respiratory disease. In lung cancer, inflammatory mediators may promote muscle protein breakdown and contribute to cachexia or sarcopenia [11], while oxidative stress, chronic inflammation, hypoxemia, and inactivity may contribute to skeletal-muscle dysfunction in COPD [32,33]. These mechanisms provide biological context for considering systemic inflammation in relation to functioning; however, they should not be interpreted as mechanisms demonstrated by the present study. The inflammation subscore was not significantly associated with any of the primary ICF categories after correction for multiple testing, and the observed associations of the ICS were predominantly driven by its lung-function component. Other factors not assessed in the present analysis, including nutritional status, muscle mass and strength, physical activity, dyspnea, and objective exercise capacity, may also contribute to functional difficulty. Larger longitudinal studies are needed to determine whether such factors mediate associations between physiological measures and ICF-defined functioning.

### 4.4. Domain-Specific Findings and Clinical Meaning

The associations with energy and drive functions and exercise tolerance suggest that the ICS may reflect part of the functional burden related to initiating and sustaining activity. However, the ICF qualifiers used in this study are ordinal clinical ratings and are not interchangeable with objective measures such as the six-minute walk test, cardiopulmonary exercise testing, accelerometry, or validated fatigue scales. These findings therefore support interpreting ICF qualifiers alongside performance-based assessment rather than as a replacement for it. The associations with walking, daily routine, and housework extend the findings from body functions to everyday activity limitations. These activities depend on integrated respiratory, cardiovascular, and neuromuscular capacity and may therefore reflect the combined effects of respiratory impairment, fatigue, and deconditioning. However, the present cross-sectional associations do not establish that the ICS can identify specific rehabilitation needs or guide individualized intervention. The association with recreation and leisure extends the findings to participation, an important dimension of ICF-defined functioning. Participation is influenced not only by physical capacity but also by personal and contextual factors. The observed association therefore suggests that the ICS captures only part of the burden related to reduced participation. The absence of an association with emotional functions reinforces the multidimensional nature of functioning in NSCLC. A previous analysis of the same cohort identified relationships between oncological biomarkers and emotional functioning [34], suggesting that different biological and clinical factors may relate to different ICF domains. Pain is similarly multifactorial and may reflect tumor-, treatment-, or musculoskeletal-related mechanisms [35]. Evidence from other rehabilitation populations also indicates that emotional outcomes may change with rehabilitation; for example, structured physical rehabilitation in patients with post-COVID condition was associated with improvements in anxiety and depressive symptoms [36]. Emotional functioning and pain should therefore be assessed directly rather than inferred from the ICS. Environmental Factor findings require particular caution and are reported as exploratory in Supplementary Materials. As described in Section 2.5, these categories were evaluated using a study-defined 0–4 problem-severity rating rather than the standard ICF barrier/facilitator qualifier; therefore, the resulting ratings should not be interpreted as equivalent to standard ICF Environmental Factor qualification. Missingness was also informative: patients for whom e115 could be rated had greater overall functional difficulty than those for whom it was coded “not applicable” (summary index 2.48 vs. 1.36; *p* < 0.001) and lower oxygen saturation (93.6% vs. 96.0%; *p* = 0.008), whereas the ICS did not differ between groups (*p* = 0.29). Thus, the e115 association was estimated in a selected, more impaired subgroup. In addition, cut-specific odds ratios for e115 ranged from 136 to 3.4, indicating violation of the proportional-odds assumption, while e120 contained only one observation in its highest qualifier level. These exploratory findings require replication in cohorts with broader applicability of these categories and explicit distinction between qualifier 0 and “not applicable.”

### 4.5. Implications for Rehabilitation Assessment and Future Research

The main contribution of this study is the demonstration that routinely collected clinical data can be linked with an ICF-based description of functioning at rehabilitation entry. The observed associations suggest that a less favorable pulmonary–inflammatory profile may accompany greater difficulty in several areas of daily life, although incremental clinical utility over simpler measures has not been established. In particular, the study does not demonstrate that the ICS performs better than individual spirometric variables, the lung-function subscore, or direct functional assessment. Accordingly, the ICS should not currently be used as a stand-alone screening, diagnostic, triage, or decision-support tool. Comprehensive pulmonary rehabilitation assessment should continue to include symptoms, exercise performance, nutritional and psychological status, comorbidities, patient goals, and contextual barriers. Moreover, no clinically meaningful ICS threshold or cut-off has been established, and the observed continuous associations should not be used to classify individual patients into clinical risk or severity categories. A critical next step is to determine whether the ICS provides information beyond simpler measures, by comparing models based on individual spirometric variables, on each subscore separately, and on the complete score. Because the score was constructed and evaluated in the same relatively small cohort, such a comparison requires internal validation and was outside the scope of the present analysis.

Longitudinal studies are also needed to assess responsiveness to rehabilitation and whether baseline or changes in the ICS are associated with objective functional and patient-reported outcomes. External validation should examine its performance across relevant clinical characteristics, including COPD status, cancer stage, treatment phase, and rehabilitation setting. Clinically meaningful thresholds or decision-support applications should be considered only if incremental value and reproducibility are demonstrated.

### 4.6. Strengths and Limitations

Strengths include consecutive recruitment, histological confirmation of NSCLC, standardized spirometry, use of readily available inflammatory markers, correction for multiple testing, and assessment within an ICF-based rehabilitation framework. Estimates were generally consistent across correlation and regression analyses, although these approaches used the same exposure and overlapping outcomes and therefore do not constitute independent corroboration. Several limitations should be considered. The sample was small, predominantly female, and recruited from a single center, limiting precision and generalizability. The cross-sectional design prevents conclusions about causality, directionality, or responsiveness to rehabilitation, because it included only the initial evaluation, so the results cannot show whether the ICS or the ICF profile changed after pulmonary rehabilitation. The analysis did not formally compare the complete score with its individual components, with individual spirometric variables, or with established clinical indices, and therefore cannot establish that combining spirometric and inflammatory information provides any advantage over lung function alone; no claim of incremental value is made. The ICS was additionally developed and evaluated in the same cohort, used equal weighting without external justification, and has not been tested for reproducibility or external validity. Residual confounding is likely because cancer stage, anticancer treatment, COPD severity, exacerbation history, comorbidity burden, anemia, nutritional status, medication use, muscle mass, physical activity, and objective exercise capacity were not included in the primary models. In particular, cancer stage and current or recent oncologic treatment status were not documented consistently enough across the available medical records to permit reliable descriptive tabulation or covariate analysis. In addition, pre-bronchodilator airflow obstruction was frequent in this cohort, but it was analyzed separately from clinically documented COPD; the study was not designed to compare patients with and without confirmed COPD. Larger multicenter studies with follow-up assessments are needed to examine whether the ICS is associated with rehabilitation outcomes and whether it can help identify patients who may require a more individualized rehabilitation plan.

Further limitations concern the ICF assessment and missing data. Only eleven categories were analyzed: eight of the 30 categories in the ICF Generic-30/Rehabilitation Set and three Environmental Factors categories. All ratings were completed by a single trained therapist, precluding assessment of inter-rater reliability. Environmental Factors had substantial numbers of “not applicable” responses, resulting in small and potentially selected samples. Pairwise deletion produced different analyzable samples across outcomes (n = 62 for seven categories, 61 for d920, 60 for e110, 31 for e115, and 19 for e120), and no imputation or formal missing-data analysis was performed. With five covariates, the ratio of observations to estimated parameters decreased from 12.4 at n = 62 to 6.2 for e115 and 3.8 for e120; moreover, e120 contained only one observation in its highest qualifier level. For the primary ICF outcomes, the adjusted ordinal models were based on 61–62 observations and five predictor terms, providing approximately 12 observations per estimated predictor parameter. Although this was more favorable than for the exploratory Environmental Factor models, the modest overall sample size and sparse counts within some qualifier levels may still have reduced model stability and precision. Accordingly, the adjusted ordinal estimates for the primary outcomes should be interpreted as exploratory and supportive rather than definitive. This concern is particularly relevant for d640 and d920, for which evidence of departure from the proportional-odds assumption was observed; the corresponding common odds ratios therefore represent average effects across thresholds and should be interpreted cautiously. For the Environmental Factor models, the substantially smaller analyzable samples remain an additional limitation, and e120 should be regarded as uninterpretable. Because the ICS was developed specifically for this exploratory analysis, its measurement properties, robustness, and reproducibility remain incompletely established. Internal consistency was not assessed, although its interpretation may be limited because the score intentionally combines complementary physiological and inflammatory domains rather than interchangeable items. Bootstrap resampling was used to obtain confidence intervals for the correlation coefficients rather than to assess score stability. Although sensitivity analyses were undertaken to assess the influence of potentially redundant components, alternative inflammation-subscore construction, and different domain-weighting schemes, these analyses do not establish an optimal score specification or weighting strategy. The equal weighting used in the primary ICS remains an exploratory analytical choice rather than an empirically validated scheme and requires confirmation and external validation in independent cohorts. Alternative standardization approaches and data-driven variable-selection or weighting procedures were not examined.

Overall, these limitations support interpreting the present findings as hypothesis-generating rather than as evidence for clinical implementation or superiority of the ICS over lung function alone.

## 5. Conclusions

In patients with NSCLC entering pulmonary rehabilitation, higher values of the exploratory ICS were associated with greater difficulty across several ICF-defined domains of functioning, including energy and drive, exercise tolerance, walking, daily routine, housework, and recreation and leisure. These associations were predominantly driven by the lung-function component, whereas the inflammation subscore was not significantly associated with any primary ICF category after correction for multiple testing. Accordingly, the present findings do not demonstrate incremental value from combining inflammatory markers with lung function. The ICS should be regarded as a hypothesis-generating research construct and not as a validated clinical instrument.

Future studies should validate the ICS in larger multicenter cohorts and determine its reproducibility, incremental value beyond lung function alone, and relationship with longitudinal rehabilitation outcomes.

## Figures and Tables

**Table 1:** Baseline clinical, spirometric-pattern, and inflammatory characteristics of the study cohort.

Characteristic	Value
Participants	62
Age, years	69.9 ± 8.87
Female sex	44 (71.0%)
Male sex	18 (29.0%)
Current smokers	24 (38.7%)
Former smokers	26 (41.9%)
Never smokers	12 (19.4%)
SpO_2_ on room air, %	94.8 ± 3.09
BMI, kg/m^2^	23.4 ± 4.16
Lung adenocarcinoma	33 (53.2%)
Squamous cell carcinoma	29 (46.8%)
FEV_1_, % predicted	62.1 ± 17.4
FVC, % predicted	76.4 ± 18.6
FEV_1_/FVC ratio	0.651 ± 0.129
Normal spirometry	9 (14.5%)
Obstructive pattern	20 (32.3%)
Spirometric pattern suggestive of restriction	12 (19.4%)
Mixed spirometric pattern	21 (33.9%)
Airflow obstruction, pre-BD FEV_1_/FVC < 0.70	41 (66.1%)
NLR	5.83 ± 4.55
PLR	252.89 ± 168.45

Data are presented as mean ± standard deviation or *n* (%), as appropriate. No values were missing for any measured variable in this table (all *n* = 62). Spirometric patterns were classified descriptively as obstructive when the pre-bronchodilator FEV_1_/FVC ratio was below 0.70 and FVC was at least 80% of predicted, suggestive of restriction when FVC was below 80% of predicted and the ratio was 0.70 or above, mixed when both criteria were met, and normal when neither was met. A reduced FVC with an FEV_1_/FVC ratio ≥ 0.70 was interpreted only as a spirometric pattern suggestive of restriction because lung-volume measurements were not available to confirm restrictive ventilatory impairment. A reduced FVC with an FEV_1_/FVC ratio < 0.70 was described as a mixed spirometric pattern for descriptive purposes only and was not considered confirmation of a mixed obstructive–restrictive ventilatory defect. The four categories are mutually exclusive.

**Table 2:** Prespecified ICF categories: eight from the ICF Rehabilitation Set (Generic-30 Set), forming the primary analysis set, together with three Environmental Factors categories analysed as exploratory.

ICF Component	Code	Category
Body Functions	b130	Energy and drive functions
Body Functions	b152	Emotional functions
Body Functions	b280	Sensation of pain
Body Functions	b455	Exercise tolerance functions
Activities and Participation	d230	Carrying out daily routine
Activities and Participation	d450	Walking
Activities and Participation	d640	Doing housework
Activities and Participation	d920	Recreation and leisure
Environmental Factors	e110	Products or substances for personal consumption
Environmental Factors	e115	Products and technology for personal use in daily living
Environmental Factors	e120	Products and technology for personal indoor and outdoor mobility and transportation

**Table 3:** Problem-severity rating used for the analysed ICF categories.

Qualifier	Problem Level	Description	Extent of Problem
0	No problem	none, absent, negligible	0–4%
1	Mild problem	slight, low	5–24%
2	Moderate problem	medium, fair	25–49%
3	Severe problem	high, extreme	50–95%
4	Complete problem	total	96–100%
8	Not specified	insufficient information	Missing in analysis
9	Not applicable	category not applicable	Missing in analysis

**Table 4:** Spearman correlations between the Integrated Clinical Score and the eight primary ICF categories (Body Functions and Activities and Participation). Benjamini–Hochberg correction was applied across these eight tests; the three Environmental Factors categories are reported in Table S4.

ICF Category	*n*	*ρ*	95% CI	*p*	BH-Adjusted *p*
b130 Energy and drive functions	62	0.38	0.15 to 0.59	0.0020	0.006
b152 Emotional functions	62	0.05	−0.21 to 0.29	0.701	0.701
b280 Sensation of pain	62	0.26	0.01 to 0.46	0.044	0.0502
b455 Exercise tolerance functions	62	0.37	0.12 to 0.58	0.0031	0.006
d230 Carrying out daily routine	62	0.37	0.13 to 0.58	0.0032	0.006
d450 Walking	62	0.39	0.16 to 0.58	0.0016	0.006
d640 Doing housework	62	0.34	0.09 to 0.55	0.0066	0.009
d920 Recreation and leisure	61	0.37	0.14 to 0.56	0.0037	0.006

BH, Benjamini–Hochberg. All coefficients and adjusted *p* values are complete.

**Table 5:** Ordinal regression of ICF category severity on the Integrated Clinical Score (effect estimates per 1-SD increase in ICS).

ICF Category	*n*	Unadjusted OR (95% CI)	Adjusted OR (95% CI)	Adjusted BH *p*
b130 Energy and drive	62	2.12 (1.28–3.52)	2.16 (1.29–3.61)	0.008
b152 Emotional functions	62	1.04 (0.67–1.62)	1.13 (0.71–1.78)	0.606
b280 Pain	62	1.50 (0.96–2.35)	1.61 (1.02–2.55)	0.047
b455 Exercise tolerance	62	1.91 (1.19–3.05)	2.02 (1.25–3.26)	0.008
d450 Walking	62	2.04 (1.26–3.31)	2.19 (1.33–3.62)	0.008
d640 Housework	62	1.78 (1.13–2.81)	1.96 (1.22–3.14)	0.009
d230 Daily routine	62	1.93 (1.19–3.11)	2.13 (1.29–3.49)	0.008
d920 Recreation and leisure	61	1.83 (1.16–2.88)	1.90 (1.19–3.03)	0.010

Adjusted models included age, sex, BMI, and smoking status; estimates are per 1-SD increase in the standardised ICS. OR > 1 indicates higher odds of a worse ICF qualifier category with increasing ICS. Benjamini–Hochberg correction was applied across the eight primary categories. The proportional-odds assumption was examined for each category. Formal testing was limited by sparse qualifier levels. For d640 and d920, evidence of departure from proportional odds was observed; consequently, the corresponding common odds ratios are shown for descriptive purposes only and should not be interpreted as constant effects across qualifier thresholds. The distribution of participants across qualifier levels and additional model diagnostics are provided in Tables S2 and S5.

## Data Availability

The datasets generated and analyzed during the current study are not publicly available because they contain clinical data from a small patient cohort and are subject to privacy and ethical restrictions. De-identified data may be made available by the corresponding author upon reasonable request, after approval by the relevant institutional/ethics committee and completion of an appropriate data-sharing agreement.

## Supplementary Materials

The following supporting information can be downloaded at: https://www.mdpi.com/article/10.3390/jcm15218211/s1, Table S1, Spearman correlations of the lung-function subscore, the inflammation subscore and the Integrated Clinical Score with each ICF category; Table S2, Distribution of participants across ICF qualifier levels; Table S3, Subgroup and interaction analyses for the association between the Integrated Clinical Score and the summary functional-difficulty index; Table S4, Exploratory results for the three Environmental Factors categories; Table S5, Assessment of the proportional-odds assumption: odds ratios for the Integrated Clinical Score estimated separately at each qualifier threshold; Table S6, Sensitivity analyses of the construction of the Integrated Clinical Score: Spearman correlations with the eight primary ICF categories and adjusted association with the summary functional-difficulty index.

## Abbreviations

The following abbreviations are used in this manuscript:

Abbreviation	Definition
ADO Index	Age, Dyspnea, and airflow Obstruction
ATS	American Thoracic Society
BMI	body mass index
BODE Index	Body-mass index, airflow Obstruction, Dyspnea, and Exercise capacity
COPD	chronic obstructive pulmonary disease
DOSE Index	Dyspnea, Obstruction, Smoking Status, Exacerbations
ERS	European Respiratory Society
FEV_1_	forced expiratory volume in 1 s
FVC	forced vital capacity
ICF	International Classification of Functioning, Disability and Health
ICS	Integrated Clinical Score
NLR	neutrophil-to-lymphocyte ratio
NSCLC	non-small cell lung cancer
PLR	platelet-to-lymphocyte ratio
PR	pulmonary rehabilitation
SpO_2_	peripheral oxygen saturation
WHO	World Health Organization
