# Supplementary material for: Exploratory Integrated Clinical Score and ICF-Defined Functioning in Non-Small Cell Lung Cancer: A Cross-Sectional Study
Source: J Clin Med. 2026 Oct 22;15(21):8211. doi: 10.3390/jcm15218211 (PMC13764500; doi:10.3390/jcm15218211)
Supplement: Supplementary file 1 [file jcm-15-08211-s001.zip › jcm-4597413-supplementary.pdf]

Table S1. Spearman correlations of the lung-function subscore, the inflammation subscore and the Integrated Clinical Score with each ICF category.

ICF category	n	Lung-function subscore	Inflammation subscore	Integrated Clinical Score
**b130 Energy and drive functions**	62	-0.39 (0.006)	+0.14 (0.468)	+0.38 (0.006)
**b152 Emotional functions**	62	-0.10 (0.462)	-0.10 (0.586)	+0.05 (0.701)
**b280 Sensation of pain**	62	-0.29 (0.024)	+0.04 (0.776)	+0.26 (0.050)
**b455 Exercise tolerance functions**	62	-0.30 (0.024)	+0.18 (0.468)	+0.37 (0.006)
**d230 Carrying out daily routine**	62	-0.34 (0.015)	+0.14 (0.468)	+0.37 (0.006)
**d450 Walking**	62	-0.34 (0.015)	+0.17 (0.468)	+0.39 (0.006)
**d640 Doing housework**	62	-0.30 (0.024)	+0.14 (0.468)	+0.34 (0.009)
**d920 Recreation and leisure**	61	-0.43 (0.005)	+0.05 (0.776)	+0.37 (0.006)
**e110 Products for personal consumption**	60	-0.25 (0.078)	+0.08 (0.552)	+0.27 (0.053)
**e115 Products/technology for daily living**	31	-0.57 (0.002)	+0.31 (0.273)	+0.59 (0.001)
**e120 Products/technology for mobility**	19	-0.34 (0.161)	+0.17 (0.552)	+0.40 (0.093)

Each cell gives Spearman ϱ with the Benjamini–Hochberg-adjusted p value in parentheses. Correction was applied within each score and within each analysis set—the eight primary categories and the three Environmental Factors categories separately. Higher lung-function subscore values indicate better lung function, so negative coefficients denote greater ICF difficulty; higher inflammation subscore and ICS values indicate greater burden, so positive coefficients denote greater difficulty. The inflammation subscore was not significantly associated with any category.

Table S2. Distribution of participants across ICF qualifier levels.

ICF category	0	1	2	3	4	Analysed	Missing
**b130 Energy and drive functions**	2	28	18	14	0	62	0
**b152 Emotional functions**	7	20	13	20	2	62	0
**b280 Sensation of pain**	2	21	19	12	8	62	0
**b455 Exercise tolerance functions**	3	14	19	14	12	62	0
**d230 Carrying out daily routine**	6	22	13	13	8	62	0
**d450 Walking**	23	17	10	7	5	62	0
**d640 Doing housework**	7	18	14	11	12	62	0

**d920 Recreation and leisure**	4	14	18	13	12	61	1
**e110 Products for personal consumption**	18	16	12	8	6	60	2
**e115 Products/technology for daily living**	7	7	7	7	3	31	31
**e120 Products/technology for mobility**	11	2	3	2	1	19	43

Qualifier 0 indicates no problem and 4 a complete problem. “Missing” counts participants coded 8 (“not specified”) or 9 (“not applicable”), which were excluded from analysis. The sparse upper levels for e120 (one participant at qualifier 4) underlie the caution applied to that category.

Table S3. Subgroup and interaction analyses for the association between the Integrated Clinical Score and the summary functional-difficulty index.

**Subgroup**	**n (group 1 / group 2)**	**Spearman ρ, group 1**	**Spearman ρ, group 2**	**Interaction p**
**Sex: female vs male**	44 / 18	+0.43 (p = 0.004)	+0.23 (p = 0.362)	0.573
**Smoking: ever vs never**	50 / 12	+0.46 (p = 0.001)	+0.18 (p = 0.577)	0.543
**Histology: adenocarcinoma vs squamous**	33 / 29	+0.43 (p = 0.011)	+0.35 (p = 0.064)	0.224
**Airflow obstruction: present vs absent**	41 / 21	+0.30 (p = 0.059)	+0.36 (p = 0.112)	0.727

No effect modifiers were prespecified; these analyses are post hoc and were not corrected for multiple testing. Interaction p values come from a product term between the ICS and the subgroup variable added to the linear model for the summary functional-difficulty index, adjusted for age, sex, BMI and smoking status (each adjusted for the three covariates other than the subgroup variable itself). Airflow obstruction is defined as a pre-bronchodilator FEV_1/FVC ratio below 0.70.

Table S4. Exploratory results for the three Environmental Factors categories.

**ICF category**	**n**	**Spearman ρ**	**BH-adjusted p**	**Unadjusted OR (95% CI)**	**Adjusted OR (95% CI)**
**e110 Products for personal consumption**	60	+0.27	0.053	1.45 (0.95–2.23)	1.52 (0.97–2.37)
**e115 Products/technology for daily living**	31	+0.59	0.001	3.47 (1.66–7.28)	4.37 (1.90–10.04)
**e120 Products/technology for mobility**	19	+0.40	0.093	2.81 (0.88–8.94)	3.21 (0.82–12.55)

Benjamini–Hochberg correction applied within these three tests. Adjusted models include age, sex, BMI and smoking status; estimates are per 1-SD increase in the ICS. These categories are reported separately from the primary analysis because “not applicable” responses left small and selected analytic samples: patients for whom e115 could be rated had a summary functional-difficulty index of 2.48 versus 1.36 in those coded “not applicable” (p < 0.001) and lower oxygen saturation (93.6% versus 96.0%; p = 0.008), while the ICS itself did not differ (p = 0.29). Results are exploratory and e120, with a single observation in its highest qualifier level, should be regarded as uninterpretable.

Table S5. Assessment of the proportional-odds assumption: odds ratios for the Integrated Clinical Score estimated separately at each qualifier threshold.

ICF category	n	OR at >0	OR at >1	OR at >2	OR at >3
**b130 Energy and drive functions**	62	—	2.22	2.79	—
**b152 Emotional functions**	62	1.85	1.34	0.90	—
**b280 Sensation of pain**	62	—	1.23	2.49	1.41
**b455 Exercise tolerance functions**	62	—	2.07	2.06	2.01
**d230 Carrying out daily routine**	62	6.05	1.60	2.39	2.52
**d450 Walking**	62	1.89	2.68	2.43	2.29
**d640 Doing housework**	62	3.73	1.29	2.66	2.22
**d920 Recreation and leisure**	61	—	1.49	2.74	1.85
**e110 Products for personal consumption**	60	1.04	2.64	1.98	1.16
**e115 Products/technology for daily living**	31	135.58	38.40	3.44	—
**e120 Products/technology for mobility**	19	2.15	—	—	—

Each column gives the odds ratio for a 1-SD increase in the ICS from a binary logistic model at that threshold, adjusted for age, sex, BMI and smoking status. Under proportional odds these should be of similar magnitude within a row. Dashes indicate thresholds with fewer than five observations on one side, which could not be estimated. A formal Brant test was estimable for only five of the eleven categories because of sparse qualifier levels; among those it indicated departure from proportional odds for d640 ($p = 0.006$), d920 ($p = 0.046$) and e110 ($p < 0.001$). For outcomes showing evidence of departure from proportional odds, particularly d640 and d920 among the primary categories, the common proportional-odds estimates are presented for descriptive purposes only. Threshold-specific odds ratios are provided to demonstrate variation across cut-points and should not be interpreted as a single constant ordinal effect.

Table S6. Sensitivity analyses of the construction of the Integrated Clinical Score: Spearman correlations with the eight primary ICF categories and adjusted association with the summary functional-difficulty index.

Score version	r with ICS	b130	b152	b280	b455	d230	d450	d640	d920	β (95% CI)	p
Primary ICS (weights 50/50)	1.00	0.38*	0.05	0.26	0.37*	0.37*	0.39*	0.34*	0.37*	0.37 (0.13–0.61)	0.003
**Leave one component out (weights 50/50)**											
Without FEV_1 % predicted	0.99	0.37*	0.01	0.23	0.36*	0.35*	0.38*	0.32*	0.35*	0.35 (0.10–0.59)	0.006
Without FVC % predicted	0.98	0.33*	0.04	0.21	0.33*	0.35*	0.37*	0.31*	0.35*	0.35 (0.11–0.59)	0.005
Without FEV_1/FVC	0.97	0.38*	0.06	0.24	0.34*	0.33*	0.36*	0.33*	0.32*	0.39 (0.15–0.63)	0.002
Without ln NLR (ln PLR only)	0.96	0.35*	0.10	0.25	0.33*	0.36*	0.40*	0.33*	0.34*	0.38 (0.14–0.62)	0.002
Without ln PLR (ln NLR only)	0.96	0.37*	−0.02	0.22	0.33*	0.32*	0.33*	0.28*	0.35*	0.35 (0.10–0.59)	0.007
**Inflammation subscore construction**											
Each blood count entered once (50/50)	0.95	0.37*	0.08	0.24	0.37*	0.36*	0.36*	0.32*	0.35*	0.38 (0.14–0.62)	0.003
**Domain weights, lung function/inflammation**											
100/0 (lung-function subscore alone)	0.71	0.39*	0.10	0.29*	0.30*	0.34*	0.34*	0.30*	0.43*	0.39 (0.14–0.64)	0.003
75/25	0.90	0.42*	0.09	0.30*	0.36*	0.40*	0.41*	0.35*	0.44*	0.41 (0.17–0.65)	0.001
67/33	0.95	0.42*	0.06	0.28*	0.37*	0.39*	0.41*	0.34*	0.41*	0.40 (0.16–0.64)	0.001
33/67	0.95	0.30*	−0.02	0.19	0.33*	0.30*	0.34*	0.30*	0.27*	0.31 (0.06–0.56)	0.015
25/75	0.90	0.25	−0.05	0.14	0.27	0.25	0.28	0.24	0.20	0.28 (0.02–0.53)	0.032
0/100 (inflammation subscore alone)	0.71	0.14	−0.10	0.04	0.18	0.14	0.17	0.14	0.05	0.18 (−0.09 to 0.44)	0.186

Each row is an alternative version of the ICS, oriented so that higher values indicate greater burden and re-standardized to mean 0 and SD 1. The 50/50 weighting is the primary ICS. r, Pearson correlation with the primary ICS. Category columns give Spearman ϱ with each primary ICF category; * Benjamini–Hochberg-adjusted $p < 0.05$ across the eight categories within that score version. β, change in the summary functional-difficulty index per 1-SD increase in the score, from the linear model adjusted for age, sex, BMI and smoking status ($n = 62$). In leave-one-out analyses, the named component was omitted and the remaining components of its domain were averaged and re-standardized as in the primary score.
